# Development of a Smart Home Interface With Older Adults: Multi-Method Co-Design Study

**DOI:** 10.2196/44439

**Published:** 2023-06-16

**Authors:** Abir Ghorayeb, Rob Comber, Rachael Gooberman-Hill

**Affiliations:** 1 Faculty of Health Sciences Bristol Medical School Bristol United Kingdom; 2 Department of Media Technology & Interaction Design KTH Royal Institute of Technology Stockholm Sweden

**Keywords:** data visualization, digital health, smart homes, older people, technology acceptance, qualitative research, mobile phone

## Abstract

**Background:**

Smart home technologies have the potential to support aging in place; however, older people’s perceptions of the value of smart homes may be influenced by their access to the information gathered by the technology. This information is needed to support their informed decision-making. Limited research has been conducted on how best to design visualizations of smart home data in keeping with the needs and wishes of older people.

**Objective:**

We aimed to investigate the design options that impact the usefulness of smart home systems, older people’s information needs, their perceptions of data visualization, and the ways they would like information displayed to them.

**Methods:**

We used a qualitative approach to empower the participants as co-designers. Data collection comprised a sequence of methods such as interviews, observation, focus groups, scenario design, probes, and design workshops. Each phase informed the next. Overall, 13 older adults (n=8, 62% female and n=5, 38% male; aged 65-89 years) consented to participate. A thematic approach was used to analyze the data set, and participants were actively involved in designing the in-home interface, which enabled them to better conceptualize their needs.

**Results:**

The information collected was clustered into 5 themes: enabling home, health, and self-monitoring; enabling opportunities for social inclusion and engagement; enhancing cognitive abilities; customizability of the display; and promoting inclusion in recreation and leisure activities. These themes informed 5 design sessions in which participants co-designed visual metaphors for the themes based on their own experiences in an age-inclusive manner. Together, the participants produced a user-friendly prototype, which they chose to call *My Buddy*. They found it useful to receive social and cognitive triggers, as well as recommendations for special diets or activities based on their mood, health, and social status.

**Conclusions:**

Smart home data visualization is much more than a nice-to-have option. Visualization is a must-have feature because it deepens the understanding of the information collected and means that technology provides information of value and relevance to older people. This may improve the acceptability and perceived utility of in-home technology. By understanding what older people want to know from smart home technology and considering how to visualize data in ways that work for them, we can provide an appropriate in-home interface. Such an interface would suggest ways or opportunities to connect and socialize; stimulate contact with close friends or family members; maintain awareness of health and well-being; provide support in decision-making, cognitive tasks, and daily life activities; and monitor health status. Older adults are the best co-designers for the development of visual metaphors that resonate with their own experiences. Our findings promote the development of technologies that foreground and reflect the information needs of older people and engage them as designers of the display.

## Introduction

### Background

Smart home technologies for older adults are becoming increasingly popular. This growth is complemented by interest in older people’s information needs as well as those of professional and family caregivers and other stakeholders. Although research into information needs often includes stakeholders, few studies have involved older people in the design process to visualize sensor data [1-6]. By working with older people to identify the information that they need and how best to view, access, and share this information, research can address the possible barriers to the adoption of smart home technology [7-9]. Appropriately designed data visualizations can help older people make the best use of smart home technologies and promote their engagement in health care and social activities [7,10].

Most studies related to data visualization from monitoring technologies have only included researchers or clinicians [2,11-15]. Many older people recognize the value of data visualizations for personal use to track changes in health and wellness and to support their decision-making and cognition [7,16-18]. However, many older people do not have a priori knowledge of the types of data collected or what it might mean for their health and well-being [18].

Table 1 presents a selection of studies that address data visualization for older people as primary users. Most studies include visualizations that were designed by researchers or technical staff and were then evaluated to determine the usability and usefulness of the interface [19], sometimes based on findings from interviews or focus groups [1,3,18]. Data visualizations have been assessed through questionnaires [19], interviews [2,3,18-21], and focus groups [14,21,22] with older people. Although visualizations have shown potential to support older people in their daily activities, the usability of the display has been questioned. Concerns about usability relate to the ease of use and effectiveness of the interface in terms of the provision of information to users and facilitation of user interaction with the system. This suggests that such interfaces may have been challenging to use or failed to meet user requirements.

**Table 1:** A summary of a selection of references.

Study author, year	Designed by researchers	Evaluated by older people	Information presented to older people	Participants	Evaluation methods
Le et al [2], 2018	2 visualizations	Limited understanding of data	Health visualization	21 older people	Semistructured interviews
Reeder et al [3], 2014	3 visual displays	Useful for caregivers, no information about how data are useful for older people	Activity level, fall scenarios	7 older people	Semistructured interviews
Ayoade et al [23], 2013	2 visualization tools	N/A^a^	Home rehabilitations	5 people who have fallen and 6 patients with knee replacements	2 user studies: falls prevention and knee replacement rehabilitation
Mynatt et al [24], 2001	In-home interface	Complex design	Communication	1 grandmother and 2 grandchildren	Field trial
Doyle et al [20], 2015	Visualizations Informed by interviews	Complex design, need for more information	Sleep activity, heart rate, weight, blood pressure, and activity level	7 older people	Semistructured interviews, focus groups
Le et al [1], 2014	1 sensor visualization, Informed by interviews	Very difficult to understand	Activity data	8 older people	Semistructured interviews
Castelli et al [25], 2017	2 visualizations and 1 visualization creation tool	First version: hard to understand and complex design	Energy, temperature, security, diary, and weather forecast	12 households (29 inhabitants, various ages)	3 interview studies, a design workshop, and system log data
Jo et al [10], 2021	Sensors available in the market	Complicated to understand and hard to read	Smart band and indoor air quality sensor’s display	9 older people	Focus groups
Caldeira et al [18], 2021	Informed by interviews	N/A	Activity status and heart rate Records	9 older people	Final product not evaluated

^a^N/A: not applicable.

Nonetheless, older adults find value in data visualization through self-tracking [2,3]. In a 6-month pilot study, Reeder et al [3] found that visualizations of sensor data were useful for older people with cognitive impairment and their caregivers. This resonates with findings from a study by Le et al [2], in which they investigated the utility of health visualizations and potential barriers by introducing 2 visualizations to 21 older adults, followed by a semistructured interview. Similar studies developed visualizations to engage older people in home exercises [23] and showed that visualizations improved the ability and confidence of the participants compared with a booklet. Other studies have used graphs and icons to support older people’s understanding of blood pressure measurements [13]. As part of a pilot study to evaluate the feasibility of using smart home devices to support aging in place, Choi et al [26] reported that participants were interested in accessing their activity level and environmental data to help them monitor and manage their health status. Visualization can also facilitate communication between older people, caregivers, and relatives [1,3,21,24] and offer a better understanding of health status [5].

Older people often face challenges in accessing and interpreting information, including data visualization. They may experience physical and cognitive changes that impact their information needs and ability to access such information. For instance, aging may be associated with changes in eyesight, cognition, and physical abilities, all of which should be considered in future visualization design [27,28]. In addition, in light of changes related to aging, some studies indicate that older people might find it difficult to locate specific information in complex interfaces, understand infographics, and control moving components [3,28,29]. Several studies have investigated the features of interfaces that can affect older people’s interactions with smart home technologies, such as the sliding method [30], bar graph [1], numerical representations, and button size [31,32]. Participants in such studies also expressed concerns about data visualization formats that have limited utility and can lead to data misinterpretation, and it is better to produce data visualizations that match older people’s own lives [32]. For instance, Mynatt et al [24] developed a digital portrait that comprised a visualization of the data collected from a motion sensor in a smart home with “butterfly” icons bordering the digital picture frame representing activity level of the participant. Mynatt et al [24] found that the design of the interface was too complex, as it conveyed 10 levels of information; however, the design did change activity levels of older people and triggered communication between them and their family members. Le et al [2] noticed that participants expressed interest in using visualizations as an intervention tool. In another study, Reeder et al [3] developed 3 interactive interfaces based on participants’ views of smart home technologies, and the evaluation phase revealed the need to reduce complexity and facilitate ease of use. Users found the bar charts difficult to understand, and the use of color created visual confusion for some. These findings were similar to those reported by Jo et al [10], who studied the usability of an indoor air quality sensor display, and by Castelli et al [25], who studied the usability of a set of sensor display by 29 participants.

A better understanding of older people and their home living environments is crucial to design appropriate technology for them [4]. Although certain studies concern data abstraction, to the best of our knowledge, there are no published studies focusing on the information needs of older people, such as how they would like to access the information and how to display it [4,5,18,33]. As Table 1 illustrates, when older people evaluate data interfaces, the data are often not presented in a way that is meaningful or appropriate for them. A substantial challenge in identifying and presenting these data lies in establishing tangible insights gathered from the real-life experiences of older people. For instance, older people are not usually familiar with the collected data sets that may require technical knowledge to understand. Furthermore, similar to people of any age, they might not be interested in all the information collected and need to select or customize what they want to visualize and access. Engaging older people as co-designers for creating visual metaphors derived from their culture or experience helps avoid ageism, with older adults having the option to select and customize the data to visualize, and helps to make the display appropriate to older people rather than creating a “big brother” style of approach [7]. Our research, which involved older people as coresearchers, highlights key points for consideration when designing visualizations for older people.

### Objectives

Our aim was to identify and describe information that older people think is essential for supporting them in decision-making, daily life activities, and cognitive tasks. We aimed to identify the types of data that older people want to access so that together we could design an interface to visually represent such data and thereby enhance the usability and utility of smart home technology.

## Methods

### Overview

**Figure 1:**
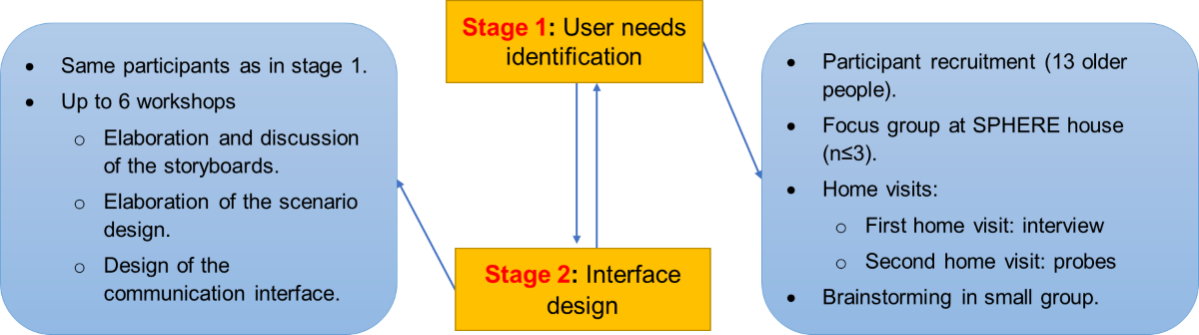
Development structure. SPHERE: sensor platform for healthcare in a residential environment.

We followed a qualitative approach in this study. We used different methods to empower the participants as co-designers and partners, as shown in Figure 1. “Co-design is meaningful end-user engagement in research design and includes instances of engagement that occur across all stages of the research process and range in intensity from relatively passive to highly active and involved” [34]. The ethics of co-design derive from an approach to community engagement that aims to go beyond passive consultation with stakeholders so that communities or groups are actively engaged. This acknowledges the importance of real-world experience in the design process. It also recognizes that as stakeholders, communities will ultimately be the most affected by design outcomes and should therefore have great influence.

We used and customized a combination of well-known participatory co-design methods to achieve our objectives. The methods comprised interviews, observation, focus groups, scenario design [35], and probes [36] (Figure 1).

### Participant Recruitment

Older people familiar with the SPHERE (sensor platform for healthcare in a residential environment) smart home technology [37] were identified and invited to participate in the study. In partnership with a local community engagement center, participants with no background in smart home technology were invited to participate. Through this partnership, we attended gatherings for older people, including an older people’s forum and dancing, knitting, and film clubs. We also placed participant information booklets in 2 public libraries, and we reached out to local organizations that support older people for assistance with the recruitment processes.

We contacted everyone who might be interested by sending out recruitment packs (invitation letters, participant information booklets, reply slips, and freepost envelopes). The aim was to include older people with a wide range of ages, education levels, health, and interests.

### Ethics Approval and Informed Consent

The study was approved by the University of Bristol Faculty of Health Sciences Research Ethics Committee (approval 57781) on November 21, 2017.

All participants provided written informed consent to participate in the study, to be audio recorded, and to have anonymous quotations and photos to be published. The participants were given the opportunity to ask and clarify any concerns before signing the consent form. A copy of the signed consent form was provided to each participant, and a second copy was kept with the researcher and held securely in university premises. Participants were made aware that they could withdraw from the study at any time without explanation or repercussions. Participants in the focus groups and workshops were provided with refreshments, and travel expenses were reimbursed. Transcripts and notes were anonymized by removing details that could lead to participant identification. The names of the participants used in this study are pseudonyms.

### Preliminary Work: Focus Groups

This work was informed by earlier findings cited in our previous work [7], in which 4 focus group sessions were conducted to investigate older people’s views and expectations of smart home technology. All participants had a positive view of smart home technology, although most participants did not recognize that they needed the technology at this point in their lives. However, the participants said that they would be willing to use such technology as they grew older or frail. In addition, participants said that they would be more receptive to the use of smart home technology if it provided access to their own health data or made it easier to participate in social events. They also wanted technology to offer new ways to have fun, shop, play web-based games, enroll in educational courses, communicate securely with others, assist family members, and fulfill other social roles. The findings of the focus groups informed our home visits and interviews.

### Home Visits and Interviews

**Figure 2:**
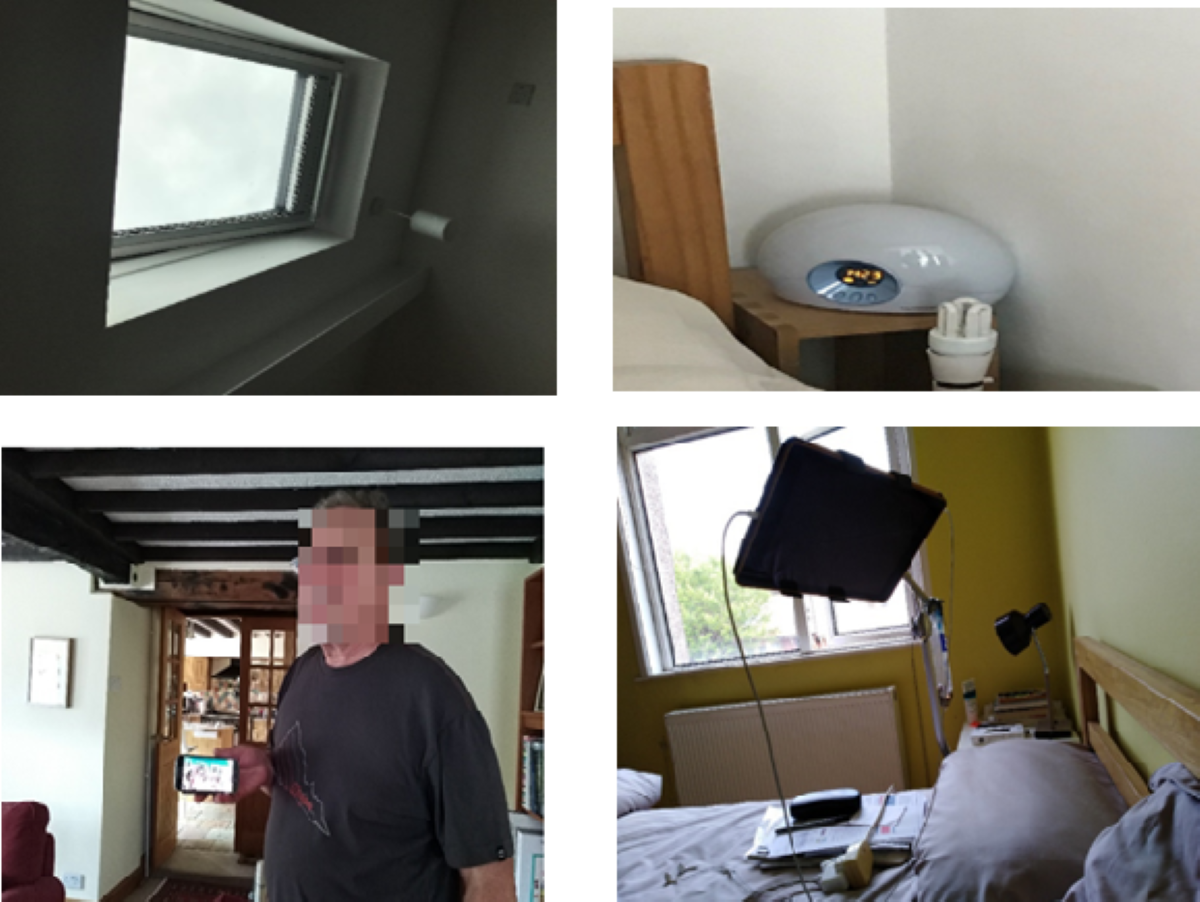
Smart gadgets at participant’s home: smart window closing when it is raining, smart alarm clock, smartphone, and tablet holder.

In total, 2 home visits were made to each participant to learn about their behaviors and attitudes toward the existing technology. The first visit comprised a semistructured interview. Discussions and observations were kept informal to reassure participants. Handwritten notes were taken, and the interviews were audio recorded. During this visit, participants were observed in their proper environment while they pointed out any item they considered particularly “smart” [38] (Figure 2). They discussed their technology use and attitudes in the context of their homes. This discussion was guided by the same topic areas. Explored topics included the use of everyday communications, privacy issues, and the identification of information needs. The first section included information about the person such as occupation, age, sex, activities, leisure, family status, the number of children and close friends, and technology use. We also attempted to learn more about the participants’ state of health, physical abilities, and particular incidents (eg, falls). In the second section, participants were asked their opinions about aging at home, smart home technology, and the reasons why older people move home. Finally, we discussed any concerns or worries that smart home technology raised and their attitudes toward being monitored and communicating remotely. The participants described activities they could no longer perform but which technology made possible and the communication issues they encountered. During the visits, the activities were flexible, depending on each participant’s abilities, interests, and capabilities.

### Probes

Cultural probe techniques [35] were used to empower the participants to share their preferences and knowledge. Cultural probes allow participants to report information about themselves and their values, thoughts, and activities. Information gathered during the first visit informed the design of our cultural probe kit, which included a camera, a diary, and questionnaires given to participants to record specific events. We began by sketching the layout of the probe on paper, ordering the questions, and leaving space for responses. Subsequently, we created and tested a prototype within the team. Our goal was to optimize the design for accessibility, attractiveness, and ease of use to ensure better results. We then conducted a trial period of 3 days with 3 participants, collecting their feedback and integrating it into the probe’s design. This included making a few questions clearer, avoiding repetition, and incorporating a scale to reflect the participants’ daily moods. After incorporating the initial feedback received, the final design revisions were made. The participants were asked to record their daily activities for 2 weeks, including their reading, listening, watching, concerns, phone use, and the types and purposes of the technology they used. We made follow-up phone calls or visits as necessary to ensure that participants understood the process and to answer any questions they may have had.

Cultural probes allowed the participants to record their lives in their own contexts and in their free time, with minimal intrusion. We gathered insights into participants’ environments that helped to identify specific issues, uncover new opportunities, and inspire the development of new design concepts. Kits were delivered to the participants in person so that each item and the overall purpose of the research could be explained. These activities were entirely voluntary, and of the 13 participants, 9 (69%) participants agreed to complete the probe kit process. They had 1 month to complete and return the documents after completing as much as they desired. Of the 9 participants, 6 (67%) completed all the elements of the probe kits.

### Design Workshops (My Buddy)

**Figure 3:**
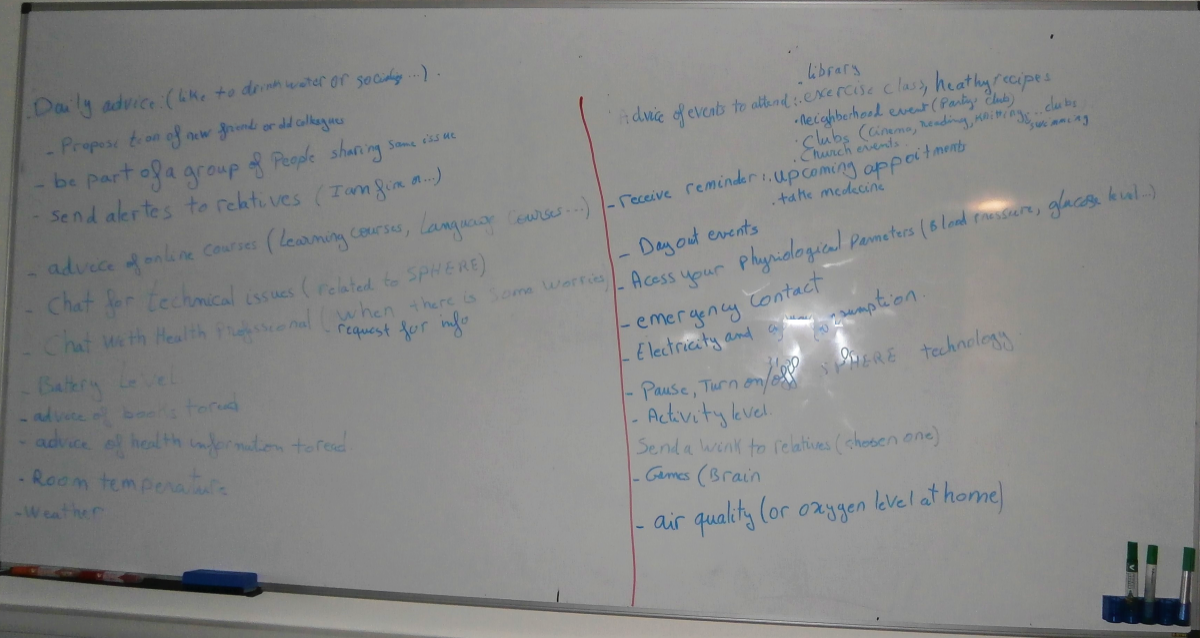
Summary of findings.

**Figure 4:**
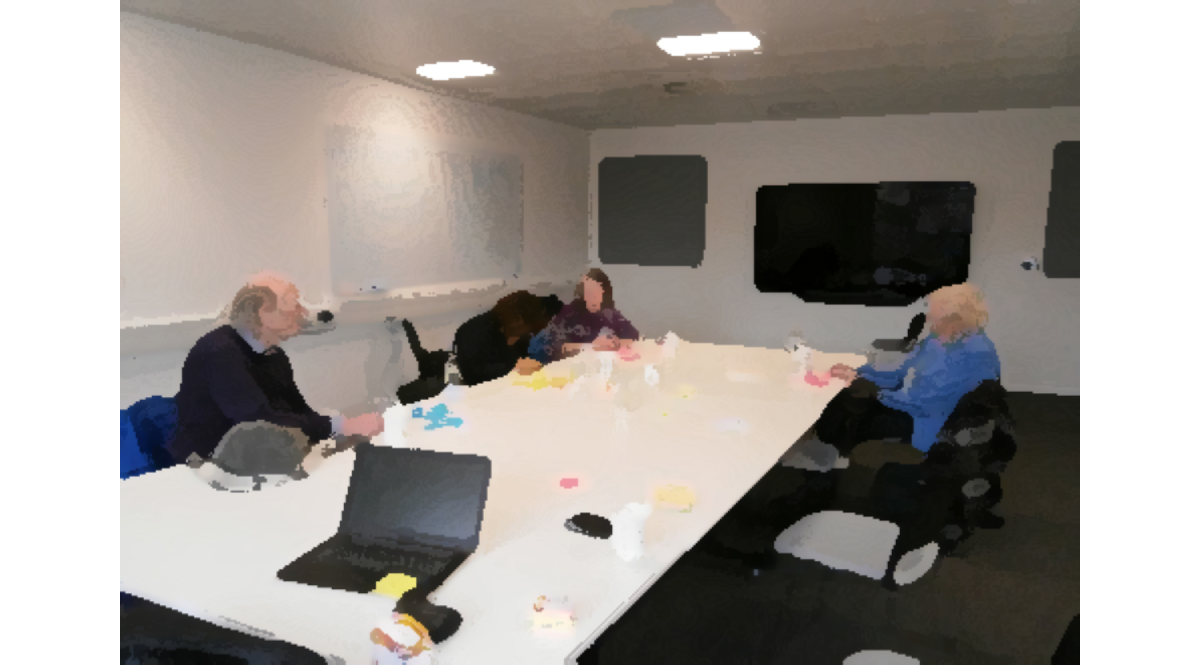
Participants using their own words to describe each functionality they wanted.

The focus group, home visits, interviews, and probe findings informed the design phase, in which we used iterative group design sessions. Of the 13 participants, 5 (38%) participants agreed to participate in the design activities. In the first session, we presented and discussed the findings of the previous phases together on a whiteboard (Figure 3) and asked the participants to identify the functionalities they would like to include in the visualization of the “in-home interface.” They were asked to write down each functionality using their own words on sticky notes (Figure 4).

**Figure 5:**
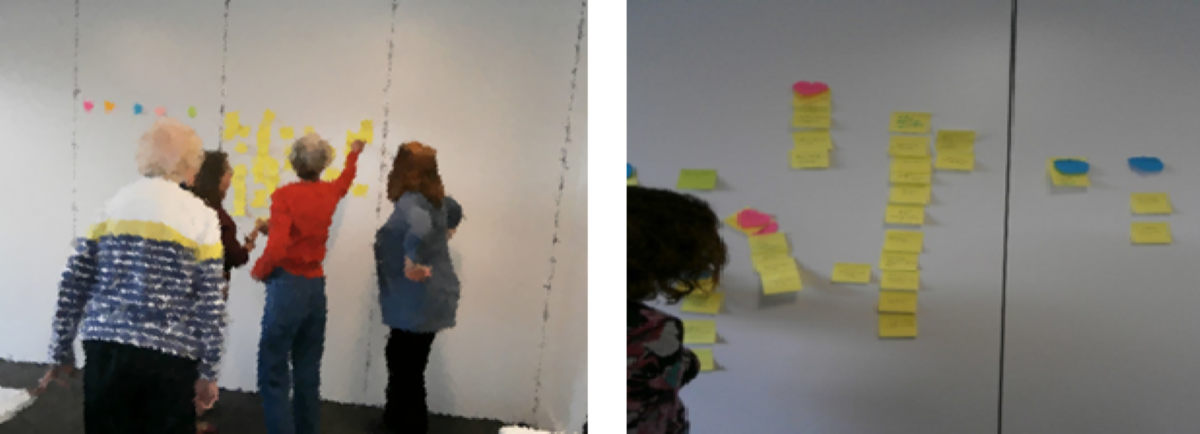
Participants organizing data.

In the second session, we presented all the sticky notes on a wall and took pictures of the participants organizing and discussing the different information, as pictures were the best way to capture the details. Participants were asked to group these items by topic and to give each topic an appropriate title. The sticky note for the title differed in color and shape (Figure 5).

**Figure 6:**
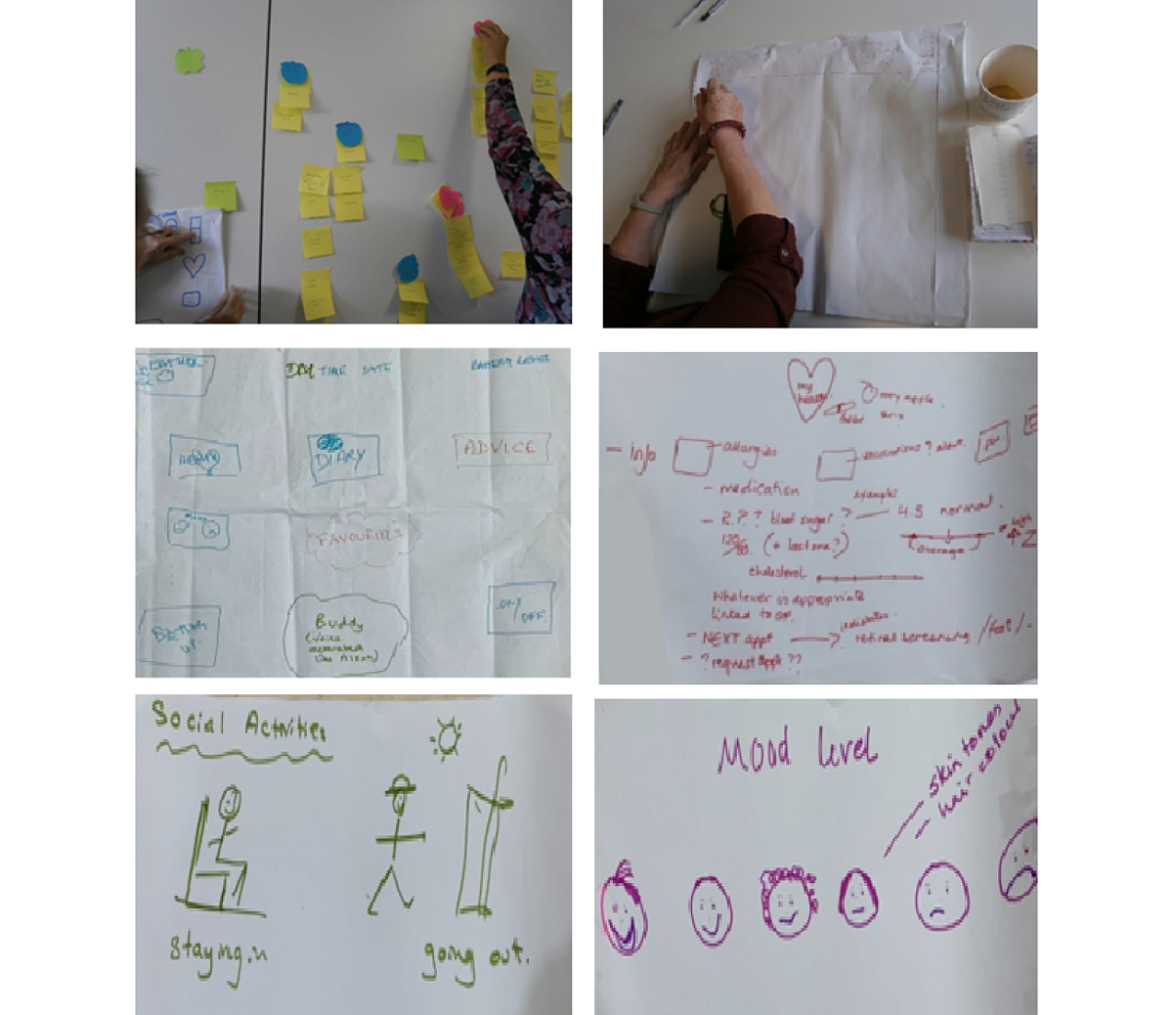
Participants drawing the main interface.

In the third session, we divided the participants into 2 groups and asked them to draw the main interface, resulting in 2 different prototypes by the end of the session. After a thorough discussion between the 2 groups, they selected 1 interface for development. The participants called this *My Buddy* (Figure 6).

**Figure 7:**
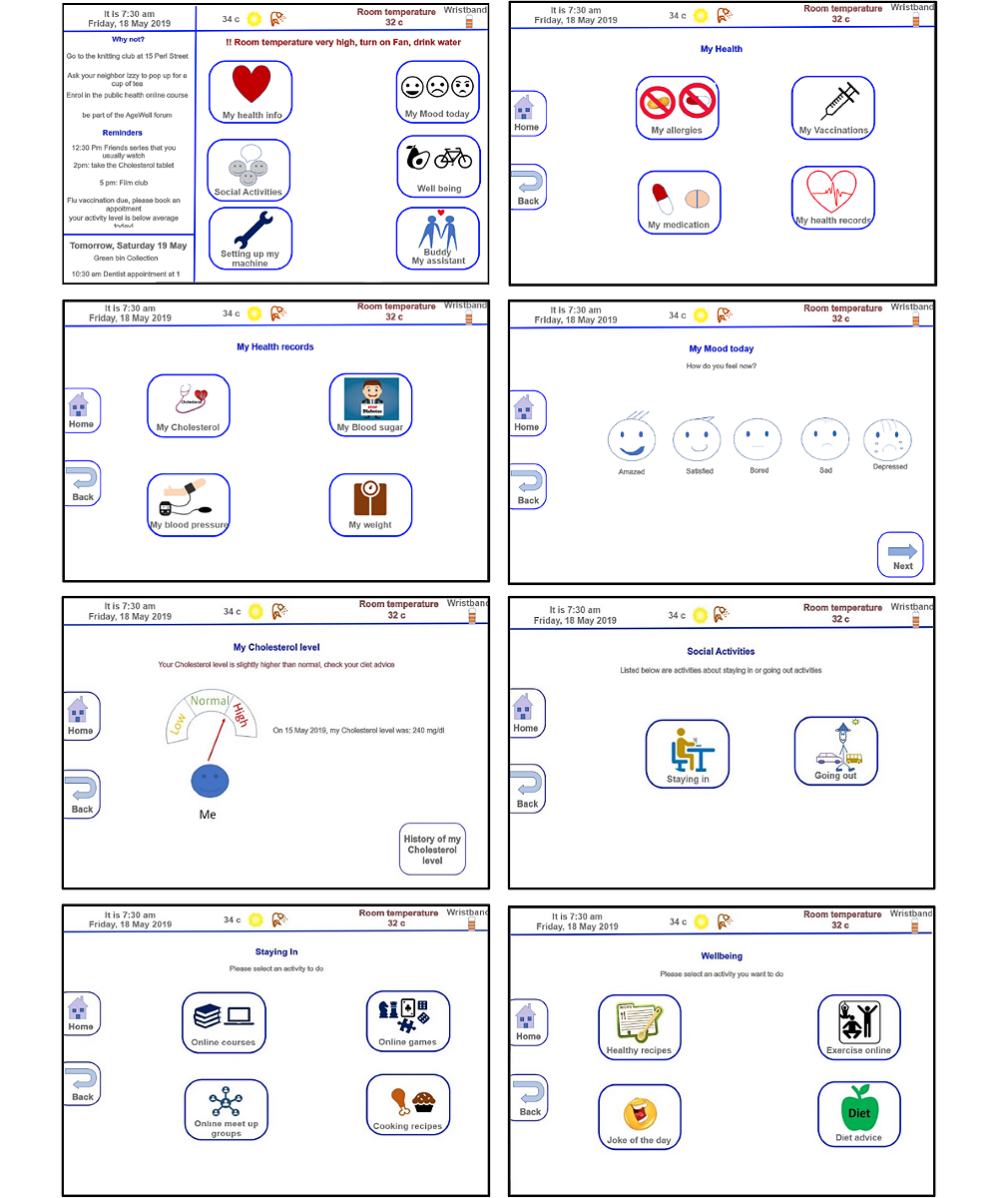
My Buddy: main and some interfaces.

In the last session, the participants evaluated the interface built using the Adobe XD software (Adobe Inc). We presented early prototypes to older people through focus groups. On the basis of these findings, we refined the interactive interface design. They suggested some improvements and proposed adding more features such as bus timetables and levels of pollen. We then developed the final version of *My Buddy* (Figure 7).

## Results

### Participants

In total, 13 older adults, aged between 65 and 89 years, with varying levels of education, health, and interests, were recruited through various channels. Of the 13 participants, 7 (54%) had prior experience using smart home technology in their homes for 8 to 12 months as part of the SPHERE project [6]. The remaining 6 (46%) of the 13 participants were members of the public who had never used smart home technology.

Table 2 summarizes the participant characteristics. Of the 13 participants, 2 (15%) participants self-identified as belonging to a minority ethnic group, and 5 (38%) participants were male. Of the 13 participants, 8 (62%) participants lived alone in their homes or apartments, whereas the rest (n=5, 38%) lived with their partners. Of the 13 participants, regarding educational background, 3 (23%) participants held school qualifications, 3 (23%) participants completed university degrees, and 7 (54%) participants had postgraduate degrees. Of the 13 participants, 2 (15%) participants had never worked before, and 1 (8%) participant was employed part time as a researcher. Most of the participants were retired but remained active and volunteered at various locations.

**Table 2:** Participant characteristics.

Participants’ pseudonym	Sex	Age (years)	Qualification
Edward	Male	73	Postgraduate qualification
Alice	Female	67	Postgraduate qualification
Bethan	Female	67	Degree qualification
Christine	Female	89	School qualification
Daniel	Male	81	Degree qualification
George	Male	81	Postgraduate qualification
Florence	Female	79	School qualification
Molly	Female	66	Postgraduate qualification
Lyam	Male	70	Postgraduate qualification
Kelly	Female	72	Postgraduate qualification
Henry	Male	68	Degree qualification
Izzy	Female	66	Postgraduate qualification
Jemma	Female	74	School qualification

### Interviews and Probes Analysis

#### Data Analysis

**Figure 8:**
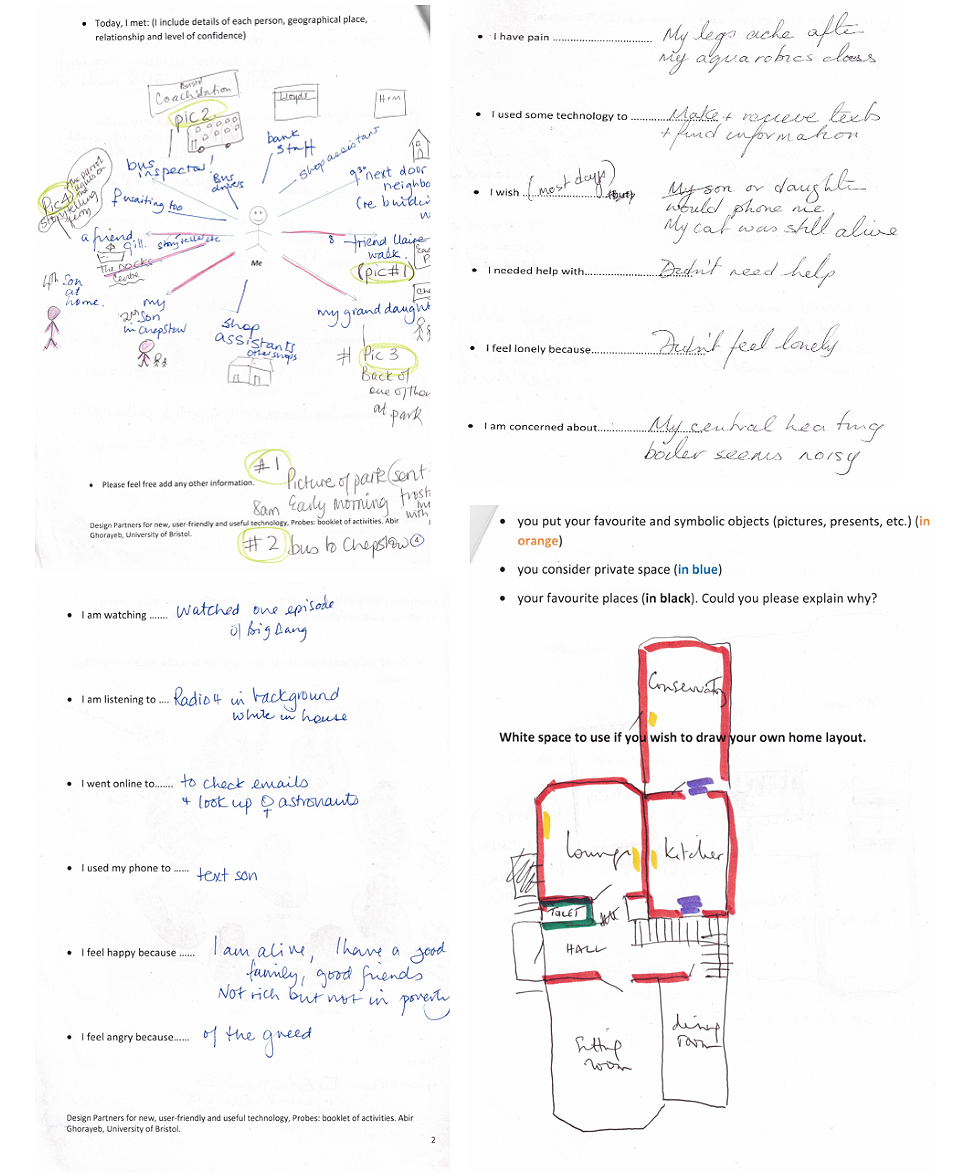
Illustrations of participants’ diary.

The probe kits produced diverse results, as illustrated in Figure 8. Initially, we read through and cleaned the probe documents and notes to remove unrelated data. Some participants provided detailed accounts of their daily routines, including their trips to stores, items they purchased, and people they met. These activities helped us gain valuable insights into their daily lives and inspired the design ideas that we explored with the participants. We then conducted an inductive thematic analysis of the interviews, probe documents, and notes, with the first and last authors collaborating on the coding and theme development. Regular data meetings were held to discuss and modify the content and analysis, including the coding and thematic development. We read and reread the transcripts, assigned codes to the data, and classified them into themes and categories [39]. As the interview feedback informed the probe questions and activities, the data analysis of one supplemented the study of the other with a number of overlapping themes.

As a result of the previous phase, most participants requested more functionalities and information from the smart home systems [7]. On the basis of the codes, we grouped the data and themes into different categories linked to enabling home and health monitoring and self-monitoring; enhancing opportunities for social inclusion and engagement; enhancing cognitive abilities; customizing the display; and promoting inclusion in recreation and leisure activities.

#### Enable Home, Health, and Self-monitoring

Participants were eager to track their health, behavior, and homes. They looked for particular details.

##### Health Parameters, Diet, and Auto-order Medication

The participants were keen to understand and manage their blood pressure, cholesterol and blood sugar levels, pulse, and activity levels. They requested results over time to help them track any changes and better manage their lifestyles. We noticed that each participant suggested what they thought was better for their health status. Some participants used the health portal to order their prescriptions:

…blood, cholesterol level and blood sugar level,...I could actually read about results for things and work out what a good blood sugar level is or not and I can perhaps do something about my lifestyle...the results of my pulse’s activity through the course of a day or through the course of a week or through the course of a month and I think for something to be perhaps meaningful, it needs to be seen in the context of time...

Henry

...[She uses the health portal] To order my prescriptions...

Kelly

...When I run out of the drugs I’m using the health portal, that goes straight to the surgery.

Daniel

Participants believed that a smart home could help them by providing a diet plan tailored to their specific health concerns. The new interface would offer an easy-to-follow recipe considering the health conditions of each person:

I’m sure there would be things around cooking.

Bethan

If I can do something about having a healthy lifestyle or improving my health, then, if it’s about diet, if it’s about exercise, it’s about things, that I guess that might improve my health or longevity or chances of remaining healthy, then I’m interested.

George

##### Enable Remote and Self-monitoring

Participants explained that they used to do some activities that they could not do anymore, such as running for the bus or going to the gym. Most participants were affected by age-related diseases and cognitive impairment. Smart home data might be used to drive behavior change, as participants wanted the system to recommend a walk, TV program, or other activities that the system indicated that they would enjoy or find helpful*:*

...like tell me how much I’ve moved all day and like how far I’ve walked or something...It is like if something had come on the telly every day and said, “Now Bethan, we’re going to do our exercises. Sit in the chair,” and I would have felt like I’d seen somebody and talked to somebody...You don’t think you’ve slept very much. Actually, it would be quite good to know, you’ve got five hours sleep or six hours sleep a day...I think things like that [monitoring steps around the home] so that you could think, “Oh, I must up it a bit,” or “Oh gosh! I’ve done well.” Could get some feedback for myself.

Bethan

Because I think from the data you could monitor your life because you don’t monitor yourself, right, and I could be sitting here watching TV for about four hours and then if you keep on looking at the data, this is what you are doing every day, you will think my God is this what I’m doing, maybe I should do something different, I should go out for a walk or something, so it’s quite important.

Molly

I would expect it to monitor my behaviour and my presence in the house or the environment...if it could choose a TV programme that it knows that I like or a radio programme which I really enjoy. So I could use something which would select things and draw my attention to them so that I could not miss a good radio programme on a Thursday morning, for instance...

Edward

If an emergency arose, all participants said that they would agree to share their data with health professionals. In total, 10 (77%) of the 13 participants agreed to share their data with trusted people only:

I mean if I was lying on the floor, and I hadn’t got my mobile phone or the landline nearby, I would want to be able to shout and know that some speaker is going to pick it up. And if there was something that you could trigger off an alarm response, then I think that would be a very good idea. I wouldn’t want to be found a week later.

Kelly

If I was being monitored for certain things by a healthcare professional of some kind, I wouldn’t necessarily want to be engaged with that. I would be happy to, abdicate responsibility and allow that information to be used on the understanding that it was for my benefit in the end anyway.

Edward

...I suppose things like Google and Alexa that would be helpful because, you might fall but you can’t move, so you could say, Alexa get help and that should be helpful. I think I would want it as part of a care package.

Izzy

Most participants wanted to keep control of the system in all cases as described in the study by Ghorayeb et al [7].

##### Enable Home Monitoring

Participants required home monitoring, as they believed that smart home technologies should accommodate common aging frailties such as lower vision, decreased mobility, increased risk of falls, and cognitive decline. They wanted to feel safer and have assistance with their daily duties. They required smart lights for dark passageways, smart plugs, and the ability to monitor who is at the entrance. They wanted the technology-enabled reminders to close an open tap or switch off the oven when necessary. They wanted to control the room temperature, air quality, and electricity consumption:

Monitor the environment itself maybe, temperature, er, yeah high temperature or low temperatures, Maybe monitor some of the appliances to see whether they were on too long or something like that...I could see whether, got a leaking tap for instance, whether someone’s left the tap on...

Edward

I think that it would be very useful if you could look at that information and say “I’m leaving too many lights on, I’m using too much electricity”...

Jemma

...The front door opening and closing is one of things that they would monitor as well as say the kettle and say the microwave or perhaps a toilet or...

Christine

I think I would expect a smart home to monitor air conditioning and turn on filters if it thought it was necessary. I would expect it if a room is not occupied and the sun is shining brightly through it and it’s in the middle of summer, I would expect it to close blinds so that the room would not get unnecessarily hot...monitor air quality and levels of background noise...air quality.

Daniel

#### Enhancing Opportunities for Social Inclusion and Engagement

##### Overview

Although social engagement has always been associated with better health and quality of life [40], it is still a real concern for older people because of the life-changing events that can be associated with aging. Although all the participants considered in-person visits as the best means of communication and interaction, they highlighted the importance of the new interface being able to suggest new ways or opportunities to connect and socialize and stimulate contact with close friends or family members. Through the probe activities, we identified the social networks of the participants, that is, the number of contacts with relatives and friends, memberships in charitable or social groups or organizations (church services), and so forth (Figure 8).

##### Communication Opportunities and Social Activities Engagement

The way that older people socialize and interact is changing, especially given their increasing use of communication technology and the role this can play in supporting social engagement. Most of the participants had already used social engagement technologies such as email, Facebook, and WhatsApp (few participants used Twitter):

I spend a bit of time doing emails and business and friends emails.

Liam

We have a little family WhatsApp. So we do often share something that will come up or they’ll send a picture...

Alicia

I either read on my Kindle or I faff about on Facebook or I might do my emails, or my bank...

Bethan

By WhatsApp, by Messenger, by email, occasionally and by telephone. I prefer it when he rings on my landline so I can sit in comfort on my sofa and I can hear better.

Kelly

Smart home technologies should promote and facilitate social inclusion by recommending social groups with similar interests. The participants suggested that smart home technologies might be useful to meet like-minded people. Older people need to feel confident, and “they have something of interest to say instead of talking about their illness all the time,” as 1 participant suggested:

...So, you could have maybe set up a group online of people in their 70s who live in this neighbourhood who have similar interests and you could maybe have a conference call or a virtual meeting for people who can’t get out, that would be very good...they’ve all got different likes, dislikes, so I think the smart home, it got to have some personal tweaks, it’s got to have a pick and mix maybe. One size doesn’t fit all, maybe.

Kelly

Maybe you’ve had the experience of having elderly relations that can only talk about their illness...but so I think is really important that we’re still able to maintain a relationship with the world. So, in that way and by the fact that you are at least in regular contact with some people online, that helps to maintain your self-confidence, which is such an important part of actually reaching out to new people...it gives you things to talk about.

Alice

However, 1 participant was resistant to the idea of contacting new people or joining new groups on the web.

##### Enhancing Family Links

Most participants were concerned that smart technologies might disturb social ties and emphasized that any new technology should enhance communication with relatives, friends, and neighbors:

The biggest concern for me is the human factor which might get stopped because of this technology. I mean so you know the children, the grandchildren they might not keep in touch with the parents.

Molly

Communication topics varied largely because the participants wanted to share their life experiences with their family members. They mainly discussed childcare, family, work and health issues, and common passions such as gardening, birds, and hobbies:

We have a little family WhatsApp. So, we do often share something that will come up or they’ll send a picture. The other day, one of the children won a little football medal so, you know, it circulated and then we all chatted a bit about how proud we were and one thing and another.

Alicia

Oh Jonny in America, oh how he is, one of my passions is square foot gardening and he does square foot gardening and I like to hear about that...

Christine

Of the 13 participants, 12 (92%) participants described their neighborhood as safe and friendly. Few of them cited a good neighborhood as the reason for their lack of need for such a system, as discussed in the study by Ghorayeb et al [7].

#### Enhancing Cognitive Abilities

##### Overview

Most participants expressed worries about their cognitive faculties. They tried to use technology to improve their abilities by enrolling in educational courses, games, reading, and web-based activities. Smart home technologies can predict users’ activity quality and promote their cognitive health by reminding them or suggesting new ways based on their usual comportment.

##### Cognitive Reminders or Enhancement

The participants suggested that the smart home interface should remind them of the day and date, time, weather, temperature, bus timetable, bin collection schedule, appointments, the location of the wearable device (if not worn at the time), and the renewing time of nearly expired cards:

With older people, it might be useful, at some point, to know what the weather is like outside because I think that does affect what people do or wear...It would be really useful if it had something on it telling me where the wearable...

Bethan

I’d quite like to know temperature change, that would be quite useful, times of the day, you know what sort of activity, intensity of activity, things like that would be quite useful...Power use, water use is quite interesting.

Edward

...Remind him that he’s now six weeks before he’s 65, so he should apply for his bus pass...it would be better if like the news in the morning said...or something came up saying, “Good morning. It’s Tuesday,” because then you’ll think “Oh Tuesday, I...”

Bethan

Smart home technologies should play a crucial role in enabling older people to monitor their daily activity levels, as this helps maintain health and achieve personal objectives. Maintaining an active lifestyle can help older people to prevent disease, lower the risk of falls, improve mental health and well-being, strengthen social ties, and improve cognitive function:

...It would be useful, at the beginning of dementia when you think you’ve got things but somebody says, “Well, actually, you didn’t turn the tap on all day today, so what have you been drinking?...chivvy them up and make sure they have a drink and whatever?” Walking...

Bethan

If you have the average levels of activity of all 70 other people that are involved or all the hundreds of people that are involved as a comparison. [Comparing between participants to challenge.]

George

Visualizing a meaningful series of data would enable older people to see the utility of this technology as one participant explained:

I would adopt this technology if I could get a meaningful series of data, then I’d consider it probably being worthwhile.

Liam

##### Enable Web-Based Learning, Cognitive Games, and Shopping

If older people have the opportunity to engage in activities and learn relevant skills, this may improve their efficacity and ability to live independently and reduce their risk of developing Alzheimer disease [41]. e-Learning and educational programs helped participants feel more confident, and brain exercises helped delay memory loss. Therefore, board games, crosswords, puzzles, reading and other types of web-based adult education classes, web-based shopping, memory games, or video games may help slow memory loss, improve daily tasks, and increase the ability to perform housework. This will also satisfy the learning and self-esteem needs of older people:

I’m very worried that I might get dementia. FutureLearn [web-based education classes], they’re usually three week, four weeks, six-week, eight week courses. So, for instance, I’ve done one about dementia, and I’ve done about 30 or 40 courses now, and they’re from all the different universities around the world and they’re free!

Bethan

Also, I’m interested in languages. I have the iPad, I watch German television...

Daniel

Watch TV, read a book, or sometimes even bake, looking for recipes...

Molly

Newspapers and books, I’ve got loads, I haven’t read all of them,...They’re all different sorts of books.

Christine

There are several apps that I quite often look at chess problems. They’re quite good actually...

Daniel

I get a newspaper and I walk back and drink my drink and do my Sudoku and things like that.

George

Web-based shopping is gaining popularity among older people at an accelerating rate. To some extent, interest in web-based shopping for groceries or clothes is defined by older people’s knowledge and experience. Being confined to the home, some older people show interest in shopping for food and clothes on the web [42]:

Living in isolated communities, showing how to use the internet and particularly for older women, the first thing they want to do is to buy clothes online as it helps give them back their self-respect...

Alice

#### Customizability of the Display

##### Overview

Older people should be given the option to select the features that they think they need or they like [7]. They should be able to select which data to visualize and who can access their data and when. The technology must be tailored to the person’s individual needs:

I think they need to be tailored to the individual person’s needs so you shouldn’t just have a one plan for everybody, although it’s smart technology it has to be tailored to the person’s individual needs...I think everybody now is gradually used to smart things in their homes, whether it’s their electricity meter or whatever, within the next five to ten years it will be normal to have certain...and I guess you could just extend that as you get older and more frail.

Izzy

...I mean obviously we get our water bill so because we’re on a meter so we know what we’re consuming...

Florence; as she had a smart meter, she was not interested to visualize information about water or electricity consumption, and she wants to omit this feature

I don’t want more technology than I need, you know, I don’t like excess technology.

Liam

Well, only if they were useful.

Bethan; when asked about data to visualize

##### Layout Consistency

Few participants stated that the display should conserve the same layout to keep them engaged, as changes to some social media displays caused them to disengage:

I have not used it much lately [about a social media tool]. I think they’ve changed the layout and I thought, oh, I can’t be bothered...I’d want to have a say in maybe designing and choosing what I’d have.

Kelly

Some participants required the ability to add information, such as to inform friends or families that they would be away for a while:

...There should be a way to say, “Well, I’m, I’m not here for a week.”

Bethan

##### Smart Home Friendly Behavior

Future systems should communicate information to older people in a friendly and easy-to-understand manner:

So, you know, I don’t want it to be too Big Brotherish but somehow...

Bethan

...make it user friendly, make it a bit larger so they can get hold of it, because they’ve got problems or issues with their fingers and thumbs...make sure that it’s functioning...

Molly

#### Promoting Inclusion in Recreation and Leisure Activities

##### Overview

Social inclusion is a complex concept for many older people, as it reflects their sense of belonging in a community where they can participate in activities based on their individual preferences. Smart home technologies should provide services and information that promote social inclusion and keep people active and occupied.

##### Old but Active: Exercises and Going Out

Coping with physical and emotional changes is particularly challenging for older people. From children moving away to the death of relatives or friends, declining health, and changes in lifestyle, participants tried to revive themselves by enjoying new things and learning to adapt to change. They tried to be involved in their community by attending local events or volunteering at different places and in different roles. They liked passing on their skills and helping other people. The participants felt young in their minds and kept themselves busy most of the week:

I don’t want to grow old being, you know, frightened of everything...Monday, Tuesday, Wednesday, Thursday, I go for a walk early with a friend who’s got a dog and Mondays and Fridays, I go to Zumba Gold and Tuesdays and Thursday, try and go to badminton and I try and make that, like, sacrosanct. I’m on several committees, so I go out on a regular basis; like once a month to this or twice a month to that; that sort of thing...I also do FutureLearn [web-based social learning platform].

Bethan

I walk a lot...I used to play tennis but I’ve got a bad shoulder, so I gave up...at the moment and all depending on the time of the year, I go quite often down to my allotment, so it’s either planting or picking...

Florence

During the day, well I usually go out somewhere, yesterday I went to a meeting at the church, today I’m going to a lunch club. Tomorrow I’m probably going to do some shopping. Thursday I’m having my hair done and do a big shop and Friday I shall wait and see what I’m going to do.

Christine

Through this study, we learned about the participants’ daily behaviors and activities. The interface should reflect their desire to lead active and engaged lives and be aligned with their self-views. Ageist attitudes are common in the community, and as age is a relative concept, we noticed that most of the participants would accept the technology much more if it did not treat them as “old”:

[When talking about his neighbor refusing to use monitoring technology] I think she thinks that it conflicts with her pride to be independent. She’s 97, you know!

Liam

##### Volunteering Activities

Among other benefits, volunteering and being active can make older people feel better. It reduces stress, prevents loneliness, and improves mood. Volunteering allows older people to maintain their physical and mental health by keeping them physically and mentally active. Meaningful and productive activities can help older people feel happier and have a positive outlook on their lives:

I’m the Treasurer to two different organisations. I’m part of the Older People’s Forum.

Bethan

I volunteer for about four different things. So, I volunteer at an international charity bookshop, half day a week. I befriend a refugee and her child, so I take them out and I visit them and so on. I volunteer at St. Georges which is a music venue...and I volunteer at a retirement home, I do a coffee morning and the poetry group. So that’s quite a regular thing.

Kelly

I help out as a volunteer, trying to help people learn either rudimentary computer skills or how to use software that they have with phones and tablets and laptops and the other commitment I have in the course of a week is I have been spending a day or at least part of a day at a community farm, where we attempt to grow organic produce...

Henry

I work with an organisation for retired people who like to provide something for the community, so I went down and volunteered at my doctor’s surgery and I started off a tea and talk club or something for people who live on their own...So we started off with about half a dozen people who, the volunteer drivers would pick up and bring them to the pub and we’d provide tea, coffee, cakes and talk once a month only about some of the things which they might be interested in, and then we started inviting more and more people...But now we’ve got 30 people and the people who identify the people who live on their own are the doctors and nurses in the surgery, so they are the ones who get in touch with them and after they’ve agreed that we can get in touch with them, then we’ll start ringing them up and provide this service. In the last four years we’ve realised that the visits they make to the doctor’s surgery have dropped!

Molly

### My Buddy

*My Buddy*, the in-home interface, was designed and developed by older people for older people. The design sessions were extremely beneficial for exploring the needs of users. During the brainstorming session, the idea of designing a new interface for older people became clearer and more concise. Consequently, they began to propose new functions and more intriguing features. They carefully chose the system functionalities they required (Figure 7).

On the primary smart home interface, the participants found it useful to have information about the wearable’s battery level, room temperature, weather and pollen level, date, and time. On the left side of the screen, participants recommended social and cognitive triggers. These triggers would be customized based on the user’s mood, health, and social status. The system may recommend a special diet, walk, web-based courses, or TV programs. They divided the reminders into 2 sections: one for “today events,” such as going to a movie or an appointment or taking medication and another for “tomorrow events,” such as “bin collection day” or an approaching appointment, allowing older people to get ready earlier. Given the weather and the advance reminders, older people can plan their trips. On the right side of the main display, older people can access their health information; specify their mood for the day; select a social activity; take care of their well-being; configure the system; or contact the assistant, dubbed *My Buddy* during the design sessions.

On the “health records” screen, participants desired access to their allergies, vaccinations, medications, and health records, including their cholesterol and blood sugar levels, weight, and blood pressure. They wanted the system to recommend activities and suggestions based on their “mood today.” Participants designed and drew 5 emojis to express their moods (Figures 6 and 7); therefore, this was not a standard scale of measurement. In their health records, participants wanted to view their current and historical blood pressure, cholesterol, blood sugar, and weight readings. They preferred customized recipes, diet advice, exercise, and consultations.

Social activities were categorized as either “local” or “going out” activities. The system may recommend web-based courses, games, meet-up groups, and recipes based on the users’ preferences, health status, and mood for the day. The “going out” option would provide the user with the ability to search for social events; nature walks or activities; sports; or “the suggestion of the day,” where the system will recommend daily events, such as knitting, dancing, and book clubs. The well-being feature would provide the user with healthy recipes, jokes, web-based exercises, and diet advice. If necessary, “Buddy my assistant” would enable the user to communicate with a health professional or technical support. The “setting up my machine” option will allow the user to customize the technology by adding or removing features, configuring data access permissions, turning the system on or off, and so forth.

## Discussion

### Principal Findings

There is a lack of research on what information older people would like to visualize and how to display it on a smart home interface [1,4,43]. Data visualization helps older people to make an informed decision about their self-care [18] and adopt smart home technology [1]. Visualized data can enable older people to self-monitor and understand and reflect on their own activities, allowing them to become more active and change their behavior. In addition, older people may become more active in monitoring their health or environment. In this study, we attempted to address this gap by understanding older people’s views of information and considering individuals’ experiences and cultures. The participants played an active role in developing and designing the in-home interface. As we started this project, we had no presumptions; rather, we sought to identify the information that older people would consider useful or vital to support their daily life activities and how they would like this information to be presented.

### Main Contributions and Links With Prior Work

Although studies have investigated how collected data would be translated into data visualization, no research has focused on the information needs of older people [4]. This work supplements the existing literature by identifying the information that would enable older people to feel able to adopt smart home technologies and to visualize and access this information. The work described in this paper was informed by an earlier study [7] and individual interviews in which older people stated that smart home technology could be stigmatizing, could signify frailty, and should provide what they consider attractive and useful. Participants expressed an interest in technologies that provide customized group lessons, learning tools or classes, cognitive triggers and activities, social events, health and diet advice, and suggestions for connecting with like-minded people. A well-designed visualization that translates collected data into a usable interface should promote active engagement of older people in health care and well-being and further social communication and activities. Participants found that the collected data and visualizations could be used mainly as prompts or triggers to enhance and provoke cognition.

Some participants were willing to share some visualizations with family members or health care professionals when needed while maintaining control over the transmitted data. Monitoring an individual’s health parameters can aid in early intervention and clinical decision-making [44]. The visualization should allow users to customize the system and gradually add features that respect their preferences and abilities as their needs evolve over time [7]. This was one of the findings of Castelli et al [25], who studied data visualization for people using smart homes, not just older people.

To improve the acceptability of smart home technology among older people, it is essential to raise awareness of its usefulness and emphasize its potential to promote independence, social interaction, and safety. It is vital to “enable the user to present the image that they want to convey to others” [45]. As discussed in our previous work [7], the in-home interface should provide older people with control over transmitted data, such as the ability to turn off or temporarily pause the technology. Most participants wanted to select which data to visualize and when and who can view their data. It should provide access to their current and historical health-related data, as well as bus timetables, healthy recipes, and dietary advice. Participants wanted *My Buddy* to remind them to take their medications, as well as to provide advice related to the weather, their health status, and mood. In addition, they wanted to be able to play web-based games, access information about physical activities, and participate in gatherings and discussions with other older people. In our study, we did not suggest which data to visualize, as we prioritized the needs of the participants. Real-life metaphorical representations were suggested by users themselves. As seen in the study by Mynatt et al [24] and others [1,10,13], visualizations that are not co-designed with potential end users may be susceptible to information overload or a lack of understanding of data abstractions. For instance, 3 interactive interfaces provided by Reeder et al [3] were found to be difficult to use; the bar chart was challenging to interpret; and the use of color led some participants to experience visual discomfort. In the study by Jo et al [10], the display of an already designed interface of an air quality sensor was difficult to read and understand. In contrast, involving participants as designers helps to identify the right information to display, as shown in the work by Doyle et al [20], who developed a display based on the analysis of semistructured interviews with 7 older people. In their study, participants complained about the ambiguity of the displayed information and expressed the need for more information. This affects people’s ability to interpret data accurately [33]. Previous studies have demonstrated the potential of visualizations to affect health and well-being [2,24]. However, these studies typically did not provide participants with actionable recommendations to follow, which limited their ability to translate visualizations into meaningful behavior changes. Moreover, older people in these studies had difficulty interpreting the visualizations and translating them into actionable steps themselves [2]. In contrast, this study involved participants who not only proposed actions based on the observed data and trends but also designed the way in which these actions should be presented to the user. These recommendations on health, social, and physical activities are likely to have a positive impact on behavior and health outcomes. This “data-to-information-to-action” approach has been suggested to be particularly effective for older individuals [2].

In this analysis, we did not observe that experiences living in smart homes, educational level, and varying age affected the choices of interface design. However, the 2 participants who had no experience with mobile phones or social media technologies were among the participants who did not want to participate in the design workshop, which may highlight a need for future research to consider how best to include individuals with less experience of technologies.

### Design Insights

In this study, participants designed the in-home interface, which they called *My Buddy*, by sketching the visual metaphors that they believed would make the tool meaningful and user-friendly. It is essential to use various research methods to uncover the requirements of older people. When asked in the interview, some older people described themselves as “healthy,” despite the activity using probes indicating that they had various chronic diseases. They did not consider themselves ill if they could perform their daily tasks independently. Thus, it was important for the design of *My Buddy* to reflect the multiple ways in which older people constructed their own health—as physical, social, and cognitive. Technology that isolated aspects of this would not be welcomed. In contrast, for instance, most of the participants used their smartphones for similar purposes, such as playing web-based games, sending or checking emails, reading the news, and searching for recipes or health-related information. They anticipated that the same functionality would be provided on the smart home display, but in a “smarter” manner, where the system recommended activities, diets, social events, or cognitive triggers based on user behavior and needs. Smart home technology should promote older people’s confidence, self-esteem, personal and social acceptance, and recognition of the contributions they made in their youth. Moving our starting point from “diagnosis” to empowerment reflects the participants’ own desires about the way to live their lives in the healthiest way possible.

Our study has shown that older people must be included in the analysis and design of their own in-home interface to incorporate their contextual knowledge, preferences, and needs. Co-design methods have been incorporated in many studies to investigate older people’s perceptions of smart home technologies. However, it may be time to consider older people as designers rather than simply informers. We suggest that it is always preferable to involve end users in the design of the new in-home interface, for instance, because graphics that a designer might use, such as pie and bar charts, may not be the most accessible to the population who will use the technology [2,28,29].

Older people are ideal co-designers for the creation of visual metaphors based on their own culture or experience, and their input enhances the production of user-friendly displays that enable older people to engage in social activities, communicate with family and friends, monitor health, seek assistance when necessary, and have the option to select and customize the data visualization.

### Limitations

This study was limited by its duration and the number of users who were involved as participants. However, previous studies indicate that usability issues can be identified using a sample of 5 to 8 participants [46]. The iterative nature of our study and the details and depth achieved indicate that the findings are relevant and transferable. Future work could involve health care professionals and relatives of end users in the design process; investigate accessibility features, such as voice control and sound alerts; and explore the next steps in the design of the interface and evaluate such a system in situ. We would also encourage future research to consider how to maximize inclusion and diversity in the research design and potential participants, in terms of sociodemographic characteristics, ethnicity, sex, and gender, as well as the cognition and experience of technology. All these factors may influence design needs and engagement with displays and technology.

### Conclusions

Smart home data visualization is essential for improving the acceptability and perceived utility of smart home technologies. To the best of our knowledge, this study is the first to address older people’s need for information and perspectives on smart home data visualization. To explore the information essential to supporting older people in their daily life activities and decision-making, we used a qualitative research approach with 13 participants as partners to design a user-friendly in-home interface. We conducted focus groups, semistructured interviews, home visits, and probe activities. From the thematic analysis of the collected data, we extracted key themes related to older people’s behaviors and interests, and these informed the design sessions. Presenting data to older people may offer them the opportunity to engage in social activities, make timely adjustments to their actions, contact relatives or friends, monitor their health status, and ask for help when needed.

Our work highlights key points for consideration when designing visualizations for older people, who were involved as co-designers in this study. The in-home interface was created by older people to present the data in an easy and meaningful way. Participants provided detailed feedback to guide improvements in the graphical user interface, content, and design changes to increase the usefulness of smart home technologies. They were interested in visualizing data about their health records and activity levels to control their blood pressure, cholesterol and blood sugar levels, pulse, and activity levels. They asked for results over time, which would help them to track any changes and to manage their lifestyle better. An in-home interface that offers people the chance to select the features needed; add further features in the future; visualize cognitive triggers and customized health, social, and diet advice; and offer communication and social engagement opportunities would be essential for future smart home technology adoption. Data visualizations should support their well-being by promoting social engagement, enhancing cognitive abilities, and enabling inclusion in recreation and leisure activities. Finally, our findings may be used to inform smart home technology developers and may apply well to other age groups. The results may increase the utility and the potential for acceptance and adoption of smart home technologies by older people.

## Abbreviations

SPHERE: sensor platform for healthcare in a residential environment

## Data Availability

Anonymous data related to this study are available to bone fide researchers on request to the corresponding author. In keeping with the research ethics approval received, the data will be available until May 2028.
